# Mental illness pathogenia: Anxiety disorders, an evolutive vision

**DOI:** 10.1192/j.eurpsy.2021.1624

**Published:** 2021-08-13

**Authors:** L. Carpio Garcia, C. Martín Villarroel, M. Sánchez Revuelta, J. Matsuura, J. Dominguez Cutanda, E. García

**Affiliations:** Psychiatry, COMPLEJO HOSPITALARIO DE TOLEDO, Toledo, Spain

**Keywords:** Evolutive Psychology, Anxiety, neurobiological development

## Abstract

**Introduction:**

There are many authors that follow and develop Pinel-hypotheses about unitary psychosis, joining recent discoveries in neuropathology and neurochemistry, supporting the vision of mental illness as neurodevelop disorders. The classification they suggest, distinguishes early, late neurodevelop disorders, and those related to traumatic factors, what determine an evolutive vision of this pathology. In terms of anxiety symptoms/disorders, they have been usually associated with categorical pathology, and treated focus on symptoms,unfortunately relapses are very frequent.

**Objectives:**

Proving that the evolutive vision may ease a change on the intervention of anxiety disorders, that would propound different therapeutic alternatives.

**Methods:**

A bibliographic search was performed from different databases, showing throw aspects related to main etiopathogenic theories about anxiety disorders from an evolutive vision.

**Results:**

Evolutive-Psychology raises that anxiety is a concomitant process to development, that grows progressively and is necessary to induce changes in it. However a high level of anxiety might block that process or causes alterations. In that sense, anxiety-disorders may be related to an excess of anxiety that provoke a fault in present handling mechanisms. According to classic dynamic-theories, these mechanisms are associated with defence concept, but now we can link them to neurobiological development. From this point, there exists an asymmetric neurological maturation through childhood-adolescence that translates different manifestations of anxiety along development, initially more related with external contemption and relationship with caregiver, but later with hormonal pulses, physical changes and separation from family.

**Conclusions:**

The evolutive vision allows to understand development fluctuation of anxiety symptoms along the growth process, more accurately than categorical classic tendency.

**Disclosure:**

No significant relationships.

